# Hamman syndrome in an 18-year-old male patient

**DOI:** 10.23938/ASSN.1096

**Published:** 2024-11-14

**Authors:** Laura Campos Modesto, Pedro Rodrigo Magalhães Negreiros de Almeida, Vitorino Modesto dos Santos, Fernanda Silva Bertulucci Angotti, Lílian Sousa Santana, Nathália Gadelha Costa Hentges

**Affiliations:** 1 University Center of Brasilia (UNICEUB). Brasília-DF, Brazil. University Center of Brasilia (UNICEUB) Brasília DF Brazil; 2 Armed Forces Hospital. Department of Medicine. Brasília- DF, Brazil Armed Forces Hospital Department of Medicine Brasília DF Brazil; 3 Master Teaching Institute President Antonio Carlos (IMEPAC). Araguari-MG, Brazil Institute President Antonio Carlos (IMEPAC) Araguari MG Brazil

**Keywords:** Diagnosis, Hamman’s syndrome, Pneumorrhachis, Pneumothorax, Diagnóstico, Síndrome de Hamman, Neumorraquis, Neumotórax

## Abstract

In 1939, Hamman’s syndrome, also know as spontaneous pneumomediastinum, was characterized by the presence of pneumothorax, subcutaneous emphysema, and pneumorrhachis. It is believed to arise from barotrauma during vaginal labor, causing alveolar membrane rupture and subsequent air leakage. Clinical manifestations are often nonspecific. Management strategies vary based on symptom severity and complications, high-concentrations oxygen therapy promoting air absorption.

We present the case of an 18-year-old male patient with a history of bronchial asthma and tobacco use, underscoring the need for heightened awareness of Hamman’s syndrome in similar cases. Prompt diagnosis and appropriate management are crucial to prevent or quickly address potential life-threatening complications. Diagnosing this syndrome in male patients can be particularly challenging, as it was initially associated with complications from vaginal labor, potentially leading to poorer outcomes.

## RESUMEN

El síndrome de Hamman se describió en 1939 como la aparición de neumomediastino espontáneo, neumotórax, enfisema subcutáneo y neumorraquis en relación a barotrauma durante el parto vaginal, con ruptura de membranas alveolares y escape de aire. Su sintomatología clínica es inespecífica. Las opciones de manejo dependen de los síntomas y complicaciones que presente el paciente, incluyendo procedimientos invasivos como descompresión y administración de oxígeno a altas concentraciones para favorecer la absorción de aire.

Para aumentar el índice de sospecha del síndrome de Hamman, presentamos el caso de un paciente masculino de 18 años con antecedentes de asma bronquial y tabaquismo. Este síndrome infrecuente debe diagnosticarse a tiempo para prevenir y controlar de manera temprana la aparición de complicaciones potencialmente mortales. Debido a su asociación con complicaciones durante el parto vaginal, la sospecha diagnóstica de este síndrome en varones es menor, lo que se asocia a peor evolución.

## INTRODUCTION

Hamman’s syndrome is a rare condition first described by Louis Hammam in 1939, characterized by spontaneous mediastinal and subcutaneous emphysema. This syndrome is often not linked with underlying lung pathology and has been associated with events such as vaginal labor or diabetic ketoacidosis. Macklin described the mechanism of the syndrome as follows: a rupture of the alveolar membranes due to barotrauma leads to the formation of a pressure gradient that allows air to escape from the lung to the mediastinum^1^^-^^10^. The Valsalva maneuver, coughing, vomiting, barotrauma, and foreign body aspiration can all contribute to the Macklin effect, in which the air leaks progressively through the pleural and pericardial layers as well as along soft tissue planes, and may also lead to the development of air leaks in the epidural space (pneumorrhachis) and intracranial spaces^1^^-^^10^.

We present the case of an 18-year-old male patient with a history of bronchial asthma and tobacco use underscoring the need for heightened awareness of Hamman’s syndrome in similar cases. This rare condition requires prompt diagnosis and appropriate management to prevent or quickly address potential life-threatening complications. Because this challenging syndrome was initially associated with complications from vaginal labor, diagnosing it in male patients can be more difficult, potentially leading to poorer outcomes. We firmly believe that single case reports can enhance general awareness, thereby reducing misdiagnosis, underdiagnoses, and unreported cases.

## CASE REPORT

An 18-year-old male patient presented to the Emergency Care Unit on June 8, 2024, with dyspnea, a severe dry cough, and chest tightness. He had a history of bronchial asthma since the age of two characterized by periodic exacerbations that responded to salbutamol, though he occasionally required hospitalization. Additionally, he had a smoking history of pack-year industrial cigarettes. One hour after receiving salbutamol, he showed symptomatic improvement and was discharged home based on his laboratory results (Table 1).

**Table 1:** Laboratory results of the 18-year-old male patient with Hamman’s syndrome

Parameters (normal values)	June 8 (at admission)	June 11	June 13
Erythrocytes (4.5-5.9 x1012/L)	6.4	6.2	6.4
Hemoglobin (13.5-17.5 g/dL)	14.7	14.3	15.0
Hematocrit (37-53%)	44.9	44.1	45.4
Mean corpuscular volume (78-100 fL)	70.2	70.9	70.4
Mean corpuscular hemoglobin (25-35 pg)	23.0	23.0	23.3
Red cell distribution width (11.5-14.5%)	15.1	14.5	14.2
Leukocytes (5-10 x109/L)	8.6	9.0	9.7
Neutrophils (40-70%)	72.1	68.5	69.8
Platelets (140-400 x109/L)	146.0	157.0	211.0
Lymphocytes (20-50%)	19.2	22.4	18.2
Monocytes (2-10%)	8.3	5.0	7.5
Eosinophils (1-7%)	0.2	4.0	4.3
Basophils (0-3%)	0.2	0.1	0.2
Sodium (135-145 mEq/L)	144.1	-	-
Potassium (3.2-5.6 mEq/L)	3.8	4.2	-
Urea (10-50 mg/dL)	40.0	30.0	-
Creatinine (0.6-1.1 mg/dL)	1.23	1.05	-
Aspartate aminotransferase (15-32 U/L)	42.0	29.0	31.0
Alanine aminotransferase (17-31 U/L)	20.0	19.0	-
Magnesium (1.7-2.5 mg/dL)	-	2.1	-
Calcium (8.5-10.5 mg/dL)	-	9.2	9.6
D-dimers (<0.5 mg/L)	-	0.63	-
Ultra-sensitive C-Reactive protein (<0.5 mg/L)	2.3	-	-
COVID-19 antigen rapid test (nasopharyngeal swab)	-	Negative	-

The following morning he experienced dyspnea accompanied by cervical subcutaneous emphysema, as well as ventilatory-dependent, intense, constant, stabbing chest pain in the left hemithorax that radiated to the back and worsened with non-productive coughing. As the subcutaneous emphysema progressed from the neck to the thoracic region, the patient was admitted to the Regional Hospital for diagnostic evaluation and treatment.

Upon physical evaluation at admission, he was tachypneic, with a respiratory rate of 24 breaths per minute. He exhibited noticeable subcutaneous emphysema in the upper third of the left hemithorax and left cervical region, along with faint crackles noted in the left hemithorax. Overall, his condition was stable: he was hydrated, non-cyanotic, with normal blood pressure (110/70 mm Hg), a regular heart rhythm (86 bpm) without heart murmurs, a Sp0_2_ of 96% on ambient air, and no wheezing. The Hamman sign (a precordial pulse-synchronous crunching sound) was absent. Table 1 presents the laboratory results.

**Figure 1:**
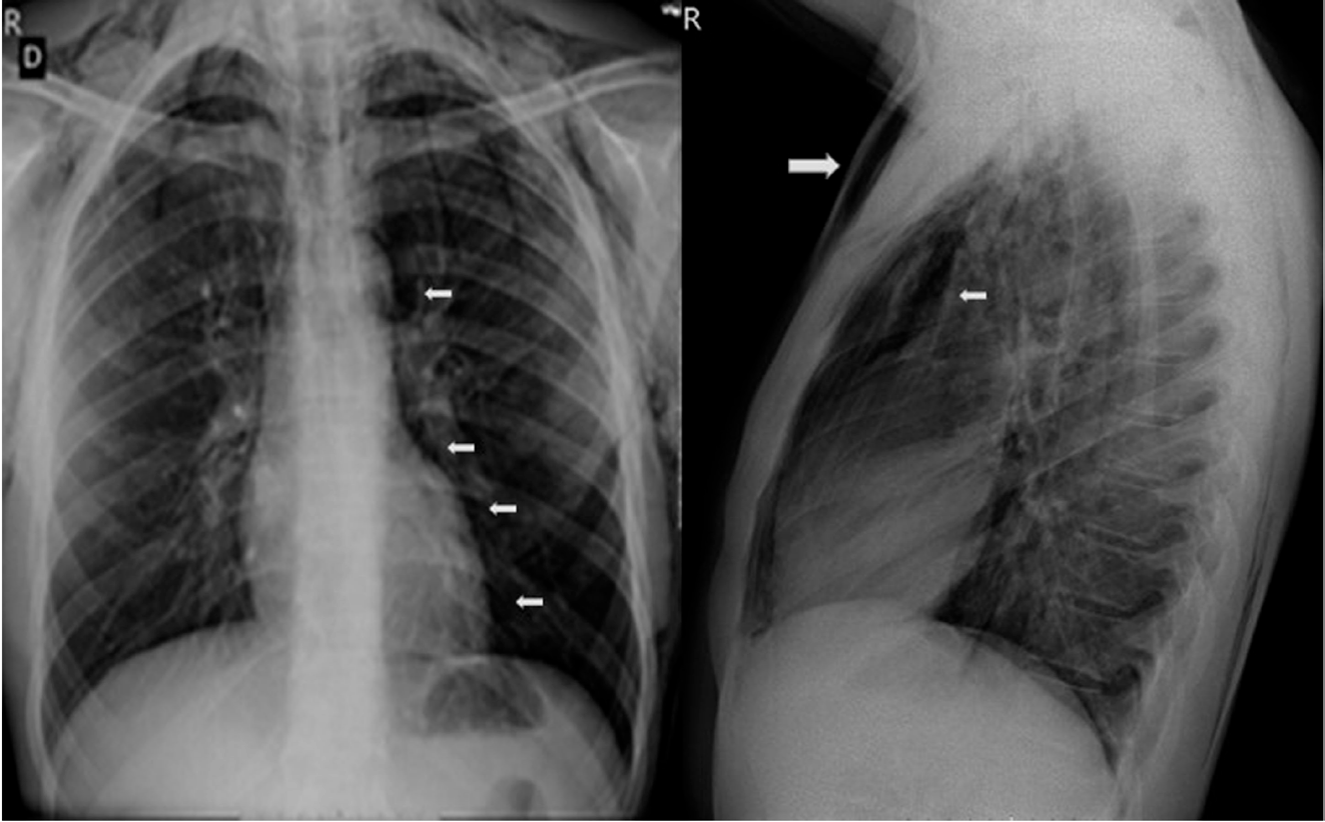
Chest X-ray at admission. **A**. Evidence of cervical and thoracic subcutaneous emphysema predominantly in the anterior region (large arrow). **B**. Pneumomediastinum extending into the deep cervical planes and pneumomediastinum (smaller arrows).

**Figure 2:**
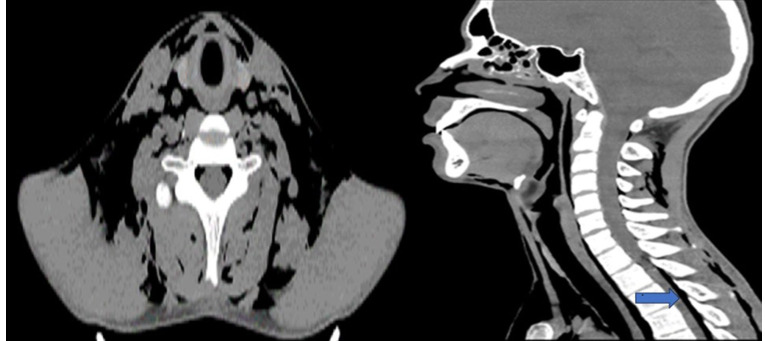
Neck computed tomography without contrast at admission. Extensive emphysema dissecting the anterior and posterior superficial and deep cervical spaces. Mucosal thickening and air-fluid levels in paranasal sinuses indicate inflammation, along with pneumorrhachis (arrow).

**Figure 3:**
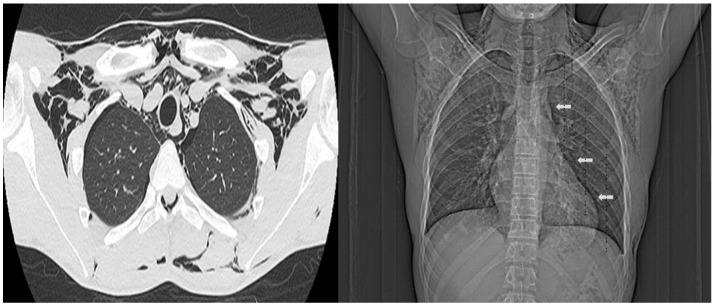
Follow-up non-contrast chest computed tomography on Day 5. Extensive emphysema is observed in the muscular and adipose planes of the cervicothoracic region, along with moderate pneumomediastinum (arrows) and minimal bilateral basal pneumothorax.

Radiographs and non-contrast computed tomography scans of the neck and chest revealed extensive subcutaneous emphysema in both the thoracic and neck regions, along with pneumomediastinum and pneumorrhachis. Additionally, there was bilateral decrease in lung transparency and the presence of scattered diffuse bands of atelectasis. In the paranasal sinuses, mucosal thickening and air-fluid levels were observed, indicative of acute inflammation (Figs. 1-2). A follow-up chest computed tomography (CT) scan on June 12 revealed extensive bilateral soft tissue emphysema in the cervicothoracic junction and chest walls, moderate pneumomediastinum, discrete gas foci within the spinal canal, multiple centrilobular ground-glass opacities predominantly in the lower lobes, and a small pneumothorax at the bases (Fig. 3). The final diagnosis was spontaneous pneumothorax and pneumomediastinum associated with pneumorrhachis, consistent with Hamman’s syndrome and the Macklin phenomenon.

The patient was counseled about the risks associated with substances that can lead to depen-dency, such as smoking, and referred to a pulmonary service for improved follow-up.

## DISCUSSION

The estimated incidence of Hamman’s syndrome is around 1 in 100,000 births, with a higher frequency observed in young primiparous women during prolonged labor^2^^,^^6^^,^^9^. Among non-obstetric cases, this rare syndrome is estimated to affect approximately one in 25,000 individuals aged between five and 34 years^10^, with about 70% of cases occurring in males in their 20s^2^. Even rarer is the occurrence of this syndrome in young children, as reported by a case involving a 17-month-old girl with no identifiable predisposing or precipitating factors. The patient achieved complete resolution within less than a week of non-invasive management^5^.

The major risk factors for Hamman’s syndrome include hyperemesis, physical exertion, chronic cough, convulsions, chronic obstructive pulmonary disease, asthma, emphysema, and history of pneumothorax, diabetes, and smoking^1^^-^^10^. Industrial and electronic cigarettes can contribute to the development of pneumomediastinum and pneumorrhachis. Additionally, smoking marijuana, cocaine, or methamphetamine can also lead to these harmful effects^1^. Excessive tongue scraping associated with violent coughing can lead barotrauma, resulting in pulmonary lesions and the development of Hamman’s syndrome^3^. Our young male patient had a history of asthma and tobacco use.

The differential diagnosis should include several serious conditions, such as tension pneumothorax, Boerhaave syndrome, pulmonary embolus, and myocardial infarction^2^.

Lung changes, such as ground-glass opacities, bronchiectasis, and consolidations, are typically seen in the lower segments of the lungs on chest radiographs and CT images^1^^-^^10^. These lesions can mimic other acute respiratory disorders.

The present case illustrates the classical manifestations and imaging findings of Hamman’s syndrome, alongside with consistent indications of recent inflammation in the paranasal sinuses. Notably, there was confirmed pneumorrhachis without evidence of pneumocephalus, and a small volume of air was present in the intraspinal spaces, which evolved asymptomatically. Pneumorrhachis may progress asymptomatically; however, in severe cases, it carries the risk of cardiopulmonary arrest and/or neurological deficits due to the central extension of pneumorrhachis through the spinal spaces, which can increase intracranial and intraspinal pressure and potentially lead to pneumocephalus^1^^,^^3^. Therefore, continuous specialized monitoring is key^1^. Our patient exhibited common manifestations of pneumomediastinum, including chest and neck pain, dyspnea, cough, and subcutaneous emphysema; however, the Hamman sign^1^ was absent. As diagnostic challenges increase in male patients lacking classic manifestations of the syndrome, it is key to employ advanced imaging techniques such as CT and MRI^1^^-^^5^^,^^7^^,^^9^^,^^10^.

Treatment may be symptomatic and/or interventional, depending on the underlying cause of the condition or its complications. Decompression or high-concentration oxygen therapy promote the absorption of air^1^^-^^10^. Our patient received symptomatic treatment. Effective follow-up of these patients necessitates a multidisciplinary approach involving various specialists.

We report this case of Hamman’s syndrome to raise awareness of health professionals and enhance their suspicious index regarding this challenging clinical condition, which can lead to potentially serious complications, particularly in patients who are not promptly diagnosed or inadequately managed.
